# Host cell surfaces induce a Type IV pili-dependent alteration of bacterial swimming

**DOI:** 10.1038/srep38950

**Published:** 2016-12-14

**Authors:** Guillaume Golovkine, Laurence Lemelle, Claire Burny, Cedric Vaillant, Jean-Francois Palierne, Christophe Place, Philippe Huber

**Affiliations:** 1 Univ. Grenoble Alpes, F-38000 Grenoble, France; 2 CNRS, ERL5261, F-38000 Grenoble, France; 3 CEA, BIG-BCI, F-38000 Grenoble, France; 4 INSERM, U1036, F-38000 Grenoble, France; 5 CNRS, USR3010, F-69342 Lyon, France; 6 Univ Lyon, Ens de Lyon, Univ Claude Bernard, CNRS, LGL-TPE, F-69342 Lyon, France; 7 Univ Lyon, Ens de Lyon, Univ Claude Bernard, CNRS, Laboratoire de Physique, F-69342 Lyon, France

## Abstract

For most pathogenic bacteria, flagellar motility is recognized as a virulence factor. Here, we analysed the swimming behaviour of bacteria close to eukaryotic cellular surfaces, using the major opportunistic pathogen *Pseudomonas aeruginosa* as a model. We delineated three classes of swimming trajectories on both cellular surfaces and glass that could be differentiated by their speeds and local curvatures, resulting from different levels of hydrodynamic interactions with the surface. Segmentation of the trajectories into linear and curved sections or pause allowed us to precisely describe the corresponding swimming patterns near the two surfaces. We concluded that (i) the trajectory classes were of same nature on cells and glass, however the trajectory distribution was strikingly different between surface types, (ii) on cell monolayers, a larger fraction of bacteria adopted a swimming mode with stronger bacteria-surface interaction mostly dependent upon Type IV pili. Thus, bacteria swim near boundaries with diverse patterns and importantly, Type IV pili differentially influence swimming near cellular and abiotic surfaces.

The early steps of pathogenic dissemination or colonization of a host involve bacteria swimming near cell surfaces. In the human body, bacteria can swim near the barriers of different body fluids, including urine, blood, lung oedema, or along the surfaces of implanted medical devices, such as blood and urinary catheters^1^,^2^. Swimming motility provides a way to travel near surfaces at much faster (~1,000-fold) rates than pili-dependent motility, and thus to cover large distances in short times. A major role of bacterial motility near surfaces is to search for nutrients through directional responses to chemical or physical cues. For pathogens, swimming also increases their chances of reaching a permissive site from where they can disseminate or form surface-attached colonies, to cause systemic or local infections, respectively. It is thus essential to investigate motion of flagellated bacteria close to host surfaces if we wish to understand the early steps of pathogenic dissemination in or colonization of a host. For this article, we studied the properties of *Pseudomonas aeruginosa* swimming near host-cell surfaces.

*P. aeruginosa* is an opportunistic Gram-negative pathogen, capable of crossing the body’s barriers in acute infections and of inducing bacteraemia. It can also form colonies on mucosae in chronic infections, such as those observed in the lungs of cystic fibrosis patients^3^,^4^,^5^. *P. aeruginosa* is a monotrichous bacterium (i.e., with a single flagellum); its polar flagellum is facultative, being present in planktonic bacteria but lost when bacteria form colonies or biofilms on surfaces^6^. The pathogenic role of *P. aeruginosa*’s flagellum has been investigated in mice using wild-type bacteria compared to mutants lacking a flagellum^7^,^8^,^9^. All these reports indicated that the flagellum is a major determinant of infection, as bacteria lacking a flagellum were much less virulent^7^,^8^,^9^. More recently, Turner *et al*.^10^ showed that flagellar motility is required for fitness and virulence in acute infection of burn wounds.

In swimming *P. aeruginosa*, the left-handed flagellar filament^11^ is powered by a reversible motor to propel the bacteria along a linear trajectory in the medium. The activity of the flagellum pushes the bacterial body forward or backward depending on the sense of rotation. The motor rotates at the same rate and speed in both directions^12^ and may be interrupted by pauses after a reverse stage, during which bacteria may abruptly change direction due to Brownian motion^13^. Hence, the bacterium can move in all directions with a “run-reverse-turn” motility pattern quite similar to the “run-reverse-flick” pattern reported for other monotrichous bacteria^14^.

As first observed for *E. coli*^15^, the motion properties of swimming bacteria are drastically modified by the vicinity of solid surfaces. One of the major changes is the increased circularity of the trajectories generated by a rotating flagellum, due to the hydrodynamic interaction experienced by the rotating body and flagellum with no-slip boundaries^16^. In parallel, the speed of swimming motion in close proximity to a glass surface was shown to be significantly reduced. In these conditions, a mean radius of the circular path of around 14.6 μm was reported for *P. aeruginosa*^17^. Differences between forward and backward motions were first observed for *Vibrio alginolyticus*^18^, and have since been reported for a range of monotrichous species^12^. Hydrodynamics predicts that the trajectory of *P. aeruginosa* forward and backward swimming near a solid surface could curve in both clockwise (CW) and counter clockwise (CCW) directions. Given bulk pattern studies previously reported^19^, the simplest extrapolated pattern of swimming near a surface is a “CW run-CCW reverse-turn” pattern.

The motion properties of swimming bacteria near complex solid surfaces, such as host-cell surfaces during infection, need to be investigated experimentally. The only observations of bacterial swimming properties on cellular monolayers, reported for peritrichous bacteria, showed no speed differences and few variations in curvature between glass and cell surfaces^20^. Conversely, experiments carried out with chemically modified glass-culture medium interfaces showed drastic modifications of the curvature of the paths^21^,^22^. These modifications could be accounted for by the modification of the hydrodynamic interactions due to partially slippery surfaces^16^. However, other bacterial structures may be involved, such as Type IV pili (hereafter pili), appendages that may influence its swimming properties close to a surface. The broad range of non-negligible biological and physico-chemical parameters and factors influencing bacterial swimming near cell surfaces prompted us to experimentally investigate the properties of *P. aeruginosa* swimming near cellular surfaces.

## Results

### P. aeruginosa swims more slowly on cell monolayers

**Figure 1:**
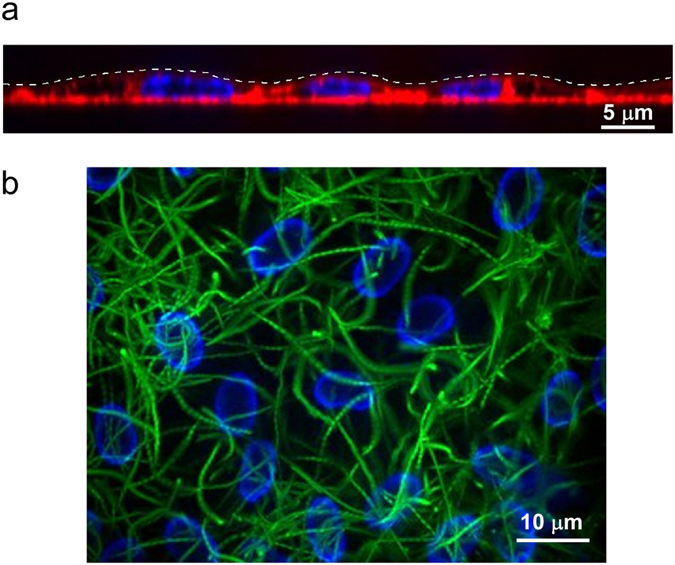
*P. aeruginosa* swimming patterns close to HUVEC monolayers. (**a**) Z-axis view of confocal microscope images showing HUVECs (plasma membranes labelled with WGA-Alexa 647 (red) and nuclei labelled with vital Hoechst (blue)). The dashed white line indicates the apical plasma membranes. (**b**) 10-s acquisition of bacterial trajectories (green) close to a HUVEC monolayer. HUVEC nuclei were labelled with vital Hoechst (blue).

**Figure 2:**
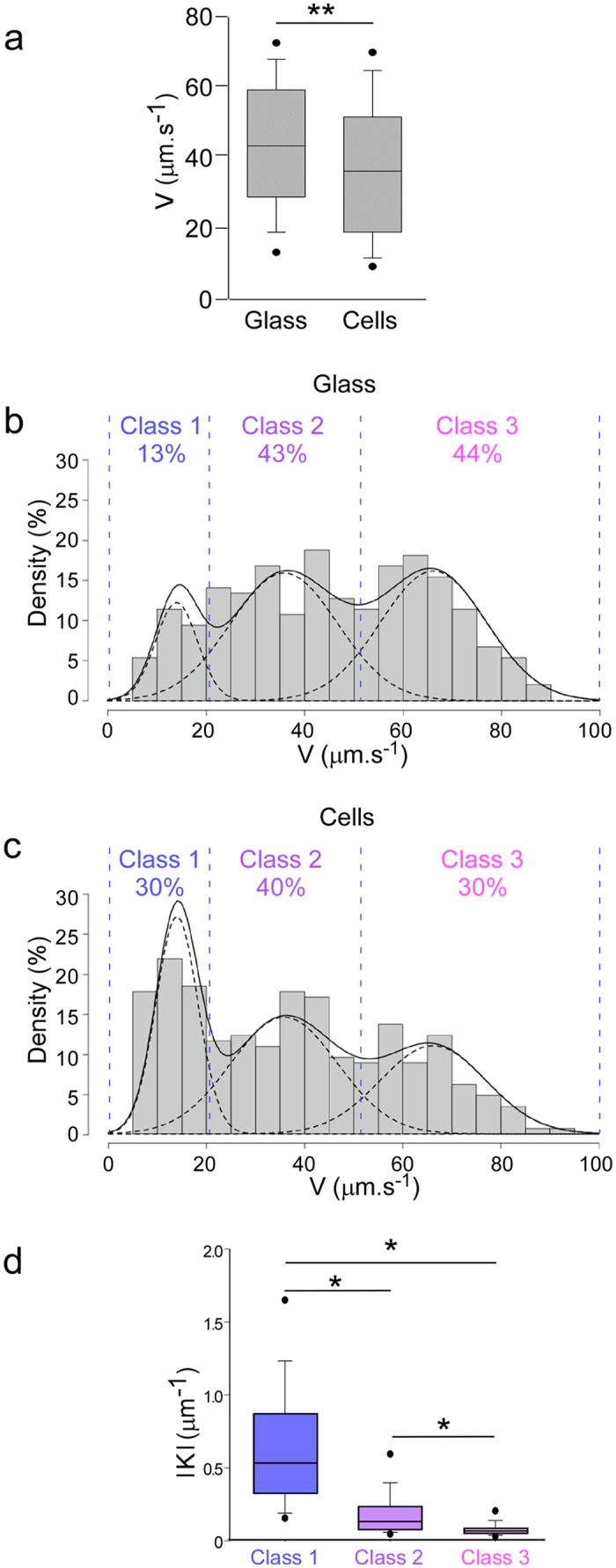
Speeds and trajectory patterns for bacteria on glass and cells. (**a**) Box plot representation of median speeds (V) for bacteria swimming in the vicinity of glass (n = 298) or cells (n = 288). The difference of medians was statistically significant (**p < 0.001) based on the Mann-Whitney’s test. (**b,c**) Histograms showing the distribution of speeds on glass (**b**) and cells (**c**). Three classes of trajectories can be distinguished with similar boundaries (blue dashed lines) for bacteria swimming on glass and on cells. The general density and class-specific density curves are represented by a continuous and dashed lines, respectively. The percentages of trajectories for each class are shown above histograms. (**d**) The absolute radii of curvature (|K|) for trajectories are shown as box plots (n = 124, 245 and 217 for Classes 1, 2 and 3, respectively). Overall comparisons using Kruskal-Wallis’s test indicates significant differences between classes (p < 0.001). Pairwise differences based on Dunn’s post-hoc test are shown: *p < 0.05.

To investigate bacterial swimming at the surface of cells, we chose cell monolayers with few topological obstacles at their apical surface to prevent steric interference with the swimming bacteria. Thus, we used primary cultures of human endothelial cells (HUVECs) grown to confluence on coverslips. HUVEC monolayers are relatively flat with shallow membrane protuberances above their nuclei (Fig. 1a). In addition, proliferation of HUVECs is limited by contact inhibition, thus protrusions owing to cell divisions are rare in this type of monolayer. For the bacterial component, we used a clinically-relevant *P. aeruginosa* strain with swimming properties, labelled with GFP to facilitate microscopic observation. Bacteria were added to the top of cell monolayers or naked glass coverslips, and microscopy images from above were recorded at 28 frames.s^−1^ for 10 s, after bleaching immobile bacteria. We used short contact times to limit as much as possible modifications of bacteria and host cells during the record. An example of bacterial trajectories close to the cell monolayer is shown Fig. 1b. It is noteworthy that bacteria swam above the HUVEC nuclei without deviation, indicating that the membrane elevation at these locations did not alter the direction of the trajectories. Trajectories of in-focus bacteria were analysed for their speed profiles. The overall speeds of bacteria swimming close to cells were significantly decreased compared to those measured for bacteria swimming close to glass (median speed values given to first decimal place: 37.8 and 45.4 μm.s^−1^, respectively; Fig. 2a). This result suggests differential interaction between bacteria and these two surfaces.

### A trimodal speed distribution

Closer inspection showed a trimodal speed distribution in both conditions (Fig. 2b,c). This finding indicates that a clonal population of swimming bacteria near a surface can follow three types of trajectories, called Classes 1–3 hereafter. It is noteworthy that the limits between classes were similar on cellular and abiotic surfaces (Fig. 2b,c), and thus the same classes can be defined for the two conditions. Class 1 was defined as trajectories with median speeds below 20 μm.s^−1^, Class 2 covered speeds between 20 and 50 μm.s^−1^, and Class 3 corresponded to speeds above 50 μm.s^−1^. Importantly, the median speeds for each class were similar between cell and glass conditions, further suggesting that these speed classes may be similar in nature on the two surface types. Compared to the applied partition, the same trajectory proportions per class were obtained using a “three-component Gaussian mixture” model (see Supplementary Table 1).

### The proportion of low-speed trajectories is higher for bacteria swimming above cell surfaces

Notwithstanding these similarities, the proportion of trajectories in each class was significantly different between cell and glass conditions, with a net increase of Class 1 at the expense of Class 3 tracks on cells compared to glass (Fig. 2b,c). Thus, the variations observed in overall speeds on glass and cells were caused by different proportions of bacteria engaged in the different classes of motility, rather than a general reduction in bacterial speeds on cell monolayers.

### Curvature correlates inversely with speed

We next investigated whether the circularity of trajectories was different between classes. The overall trajectory circularity for each class, <|K|> (Fig. 2d), was calculated by averaging the absolute curvature values for each point, <|K|>. The circularity distributions exhibited striking differences, with the highest <|K|> in Class 1 and the lowest <|K|> in Class 3, correlating inversely with the average speeds. The differences between trajectory curvature on glass and cells in each class were not significant (not shown), confirming that the decrease in average speed on cells vs. glass was caused by a redistribution of the trajectories between classes, rather than by modifications of the motion characteristics of each class.

### The proportion of linear versus curved motions is highly variable between classes

**Figure 3:**
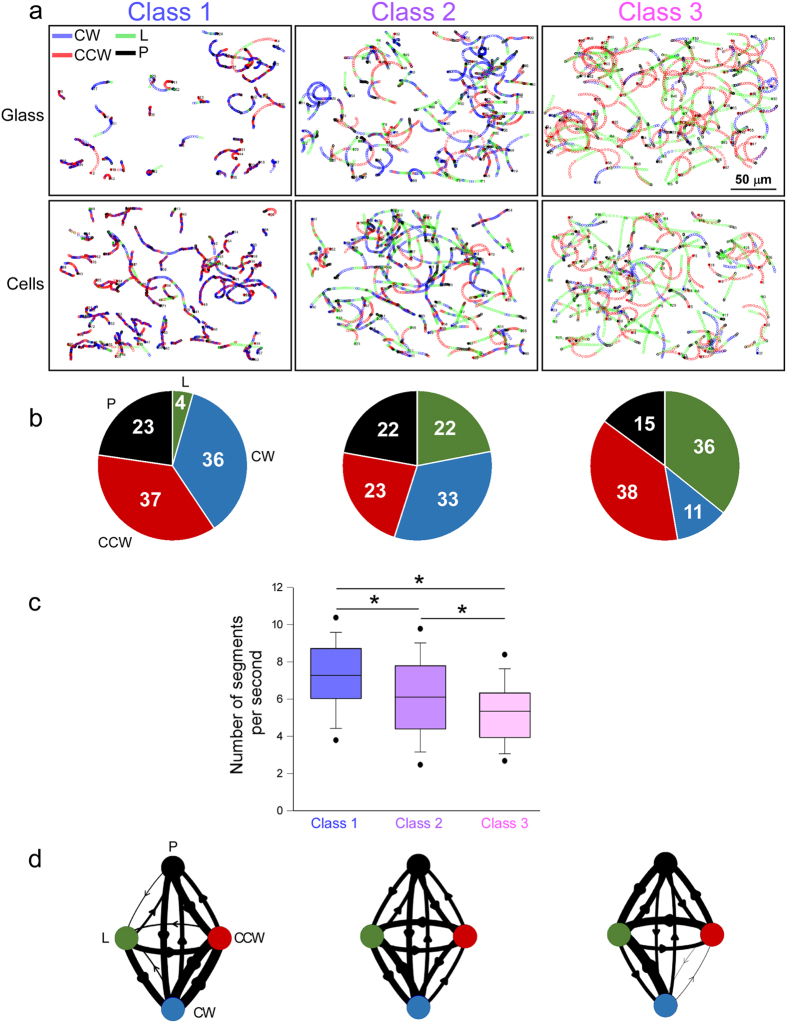
Analysis of trajectory circularity on glass and cells. (**a**) Class-specific trajectories on glass and cells. Each trajectory was divided into linear (L, green), clockwise (CW, blue) or counter-clockwise (CCW, red), and pause (P, black) segments. (**b**) Proportion of trajectories corresponding to each segment type, for each class. (**c**) The number of successive segments per time unit (s) was calculated and represented as box plots for each class (n = 124, 245 and 217 for Classes 1, 2 and 3, respectively). Overall comparisons using Kruskal-Wallis’s test indicates significant differences between conditions (p < 0.001). Pairwise differences using Dunn’s post-hoc test are shown: *p < 0.05. (**d**) Transition matrices represented as a diagram between the different section types. The thickness of the connecting lines is proportional to the number of transitions. For b-d, the trajectory parameters on glass and cells were pooled, as no significant difference could be observed when assessed independently.

Representative examples of the three trajectory classes are reported in Fig. 3a. These trajectories show curved (CW and CCW) and linear sections, as well as pauses (see Materials and Methods for the physical definitions of section types). A rapid examination of the trajectories indicated that the three speed classes corresponded to three different swimming patterns. In Class 1, alternating short and highly curved CW and CCW sections predominated, forming tight zigzag paths. In Class 3, sections were much more extended, with a majority of low-curvature CCW and linear sections. In Class 2, zigzag portions were scarce, but the pattern was more complex than in Class 3 as it also involved CW sections. Interestingly, the radii of curvature were higher for the curved sections of Class 1 tracks than for those of Class 2 and 3 tracks (see Supplementary Table 2), indicating that bacteria engaged in Class 1 trajectories turned sharper in the curved sections than in the two other classes.

The duration and sequence of the sections were specific to each of the three patterns. Indeed, the relative times spent in each section type (Fig. 3b) showed a striking increase in the proportions of linear sections as we progressed from Class 1 to Class 3 (4 to 36%, respectively). This increase in linear trajectory was at the expense of circular sections: CCW for Class 2 and CW for Class 3. The pauses were also shorter in Class 3 compared to Class 2 and Class 1, while transitions were more frequent in Class 1 than in Classes 2 and 3 (Fig. 3c). The probability of transition between section types has been summarized in a diagram for the three classes of swimming behaviour (Fig. 3d). In Class 1, the most probable transitions were between CW and CCW circular motions. In Class 2, transitions were more balanced between the four motion types. In Class 3, transitions tended to favour linear motion. In conclusion, bacteria used similar motion types (curved or linear) and pauses, but in different proportions and at different frequencies, to generate the three trajectory classes.

### Type IV pili are involved in Class 1 swimming patterns on cell surfaces

**Figure 4:**
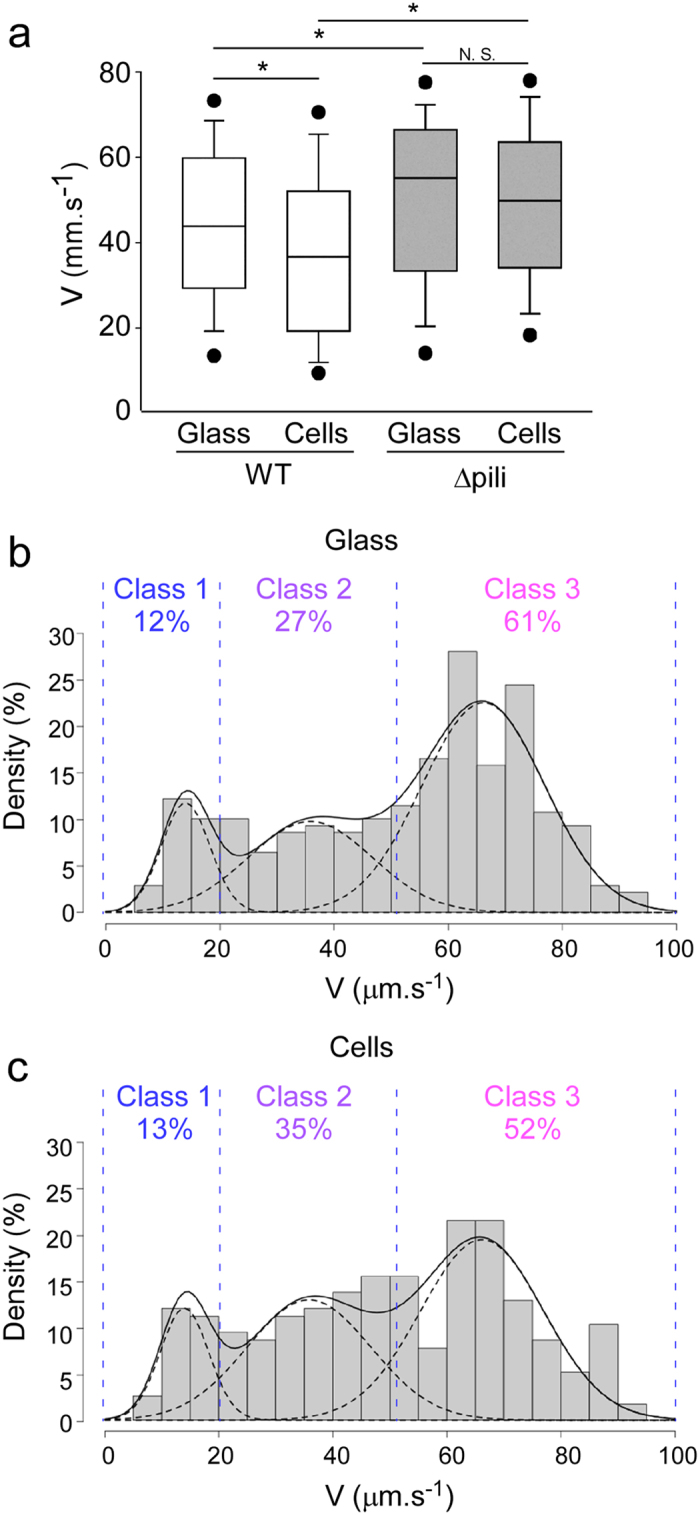
Speed distribution for trajectories of pili-deficient bacteria. (**a**) Box plot representation of speeds for Δpili (grey; n = 278 and 234 for glass and cell surfaces, respectively) and WT (white; as in Fig. 2a) bacteria. Overall comparison using Kruskal-Wallis’s test indicated a significant difference between conditions (p < 0.001). Pairwise differences using Dunn’s post-hoc test are shown: *p < 0.05; N.S., not significant. (**b,c**) Histograms showing the distribution of speeds on glass (**b**) and cells (**c**), as in Fig. 2 (**b,c)** The proportions for each class are shown above the curves.

To determine whether bacterial pili influenced *P. aeruginosa* swimming near a surface, we used a pili-deficient strain (Δpili) and examined its swimming properties on glass and cell monolayers. Overall speeds were much higher with the Δpili strain than with the wild-type (WT) strain (with median speeds of 52.1 vs. 45.0 μm.s^−1^ on glass and 50.2 vs. 37.8 μm.s^−1^ on cells; Fig. 4a). This alteration of speed resulted at least in part from a striking increase in Class 3 and a decrease in Class 2 trajectories in the absence of pili (Fig. 4b,c). These observations suggest that pili contributed to how bacteria produce Class 2 swimming trajectories, i.e., bacteria subjected to intermediate interaction with the surface.

Significantly, unlike with the WT strain (Fig. 2b,c), the proportion of Class 1 trajectories for the Δpili strain was similar on both surface types (13 and 12% on glass and cells, respectively; Fig. 4b,c). This observation indicates that the dramatic increase in the percentage of Class 1 trajectories with WT *P. aeruginosa* on cells vs. glass was pili-dependent. As a result, the overall speeds for the Δpili strain were not significantly affected by the surface type (Fig. 4a).

Altogether, we show that the major effect of pili deficiency was to modify the distribution of trajectories between classes, increasing the proportion of bacteria interacting least with the surface.

## Discussion

Swimming is a highly powerful motion through which bacteria can explore biological tissues. In this article, we show for the first time that not all swimming bacteria in a clonal population of *P. aeruginosa* respond in the same way to the presence of a surface. Three classes of *P. aeruginosa* swimming could be defined near a surface, primarily based on the mean speed of trajectories. These speeds were associated with specific swimming patterns (Table 1). Bacteria in Class 1 swim slowly and take frequent pauses. The trajectory pattern involves frequent alternation of short and curved CW and CCW motions. Class 3 encompasses bacteria that swim rapidly with rare pauses; the trajectory is more linear and uniform, and shows a significant CW-CCW asymmetry. The Class 2 pattern is quite similar to that of Class 3, but with a greater number of pauses and CW sections.

The three patterns were observed for bacteria swimming near glass and cells, with and without pili. Thus, pattern formation was not exclusively triggered by pili, or by a specific surface type. However, the proportion of bacteria adopting one of the three patterns was altered in different experimental conditions. Only the distribution between the three patterns was specific to surface-bacteria interactions. Hence, patterns must result from different types of interaction prevailing near the surface.

Hydrodynamic wall effects on bacteria swimming near a solid surface^16^ predict a decrease in the curvature (<K>) and an increase in the speed (<V>) as the distance between the bacterium and the surface grows. Thus, bacteria following Class 1 trajectories are anticipated to be very close to the surface and to interact most strongly with it. By opposition, Class 3 trajectories are associated with the bacteria furthest from the surface and displaying the weakest interaction with it. The Class 2 pattern, showing alternating “CW – CCW” sections, is theoretically expected for monotrichous bacteria swimming close to a solid surface. The newly observed Class 3 pattern could be an intermediate stage between the expected Class 2 pattern for near-surface swimming and the “in-bulk pattern”, where alternative run and reverse sections result in linear trajectories. In contrast, the hydrodynamic model cannot fully explain the Class 1 pattern. In our study, an additional friction was at least partially related to the presence of pili (see below).

**Figure 5:**
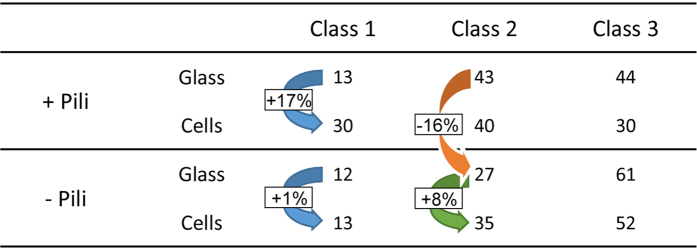
Summary of trajectory distribution as a function of surface type and the presence of pili. The blue arrows represent pili-dependent recognition of cells, the yellow arrow, pili-independent interaction with glass, and the green arrow, pili-independent recognition of cells.

In terms of host-bacteria interaction, four major conclusions can be drawn from the results presented in this paper (Fig. 5). Firstly, bacteria swam more slowly on cells because there was a higher proportion of Class 1 trajectories (13 to 30%) and fewer Class 3 trajectories (44 to 30%). Thus, bacteria interacted more closely with cells than with glass surfaces. This increased interaction on cells was exclusively mediated by pili, as the Δpili strain exhibited no significant variation in the proportion of bacteria engaging in Class 1 swimming on the two surfaces (12 and 13% on glass and cells, respectively). It is therefore tempting to speculate that pili close to cell membranes transiently interact with specific receptors, such as N-glycans, asialo-gangliosides and integrins, as previously described^23^,^24^,^25^. This reversible interaction may lead to extended pauses and a greater number of directional changes, and in general it will keep the bacterium close to the surface. Alternatively, it cannot be excluded that cell surface contact activated pili production within minutes, which would increase the global adhesive activity of bacteria. Secondly, as previously reported^17^,^26^,^27^, pili may also promote interaction with glass surfaces, as the proportion of bacteria engaging in Class 2 trajectories decreased on glass (43 to 27%) in the absence of pili. Alternatively, pili may bias flagellar rotation through direct interaction between the two organelles or by their potential alteration of bacterium hydrodynamics, which would alter speed and rotation. Thirdly, interaction of bacteria with cells is to some degree independent of pili, as the proportion of Class 2 swimmers remained higher on cells than on glass in the absence of pili (35 to 27%). This interaction could be due to receptors for the flagellum, several of which have been described on host cells^25^,^28^,^29^. The flagellum is thus another adhesive appendage potentially allowing increased interaction with cellular surfaces compared to glass. Fourthly, in the absence of pili and cellular receptors (i.e., on glass), some bacteria still adopted Class 1 and Class 2 trajectories (12 and 27%, respectively), suggesting that physical and chemical interactions with this surface are possible and promote strong or intermediate attractions. Earlier reports indicated that the flagellum efficiently interacts with glass and plastic^17^,^26^,^27^ and can be used as a surface sensor by other bacterial strains^30^. More work will be needed to determine whether this interaction is due only to the hydrodynamic forces exerted by the glass surface on the bacteria and its flagellum, or whether some chemical interaction between the surface and bacterial appendages other than pili (e.g. the flagellum) causes Class 1 or Class 2 trajectories.

In conclusion, not all swimming bacteria in a clonal population respond in the same way to a surface. Class 1 trajectories are probably helpful for local scanning of surfaces, while Class 3 trajectories are useful for large-scale investigations. Moreover, the presence of pili and the surface type both influence swimming behaviour. Recently, Persat *et al*.^31^ reported that pili are mechanical sensors that regulate virulence factor upregulation in a substrate-dependent manner. Furthermore, pili may be able to sense the local viscoelasticity of the surface^32^. Brief pili-dependent surface-bacteria interaction during swimming may thus provide the bacteria with information on the surface type, and could possibly initiate the transcription of major virulence factors.

The next step will be to understand the fate of bacteria from each class at longer time points. It will be important to determine which motion type leads to attachment, colony formation or epithelial penetration. We recently showed that the bacteria which cross the epithelial layer swim individually, patrolling above the surface, and are not those present in immobile aggregates on the surface of the cellular monolayer^33^. The pathogenic behaviour of bacteria may thus be a direct consequence of their swimming behaviour near the cellular surface.

## Materials and Methods

### Cell culture

The use of umbilical cords for scientific purposes is authorized by the L1211-2 act from the French Public Health Code. Written, informed consent was obtained from each woman who donated an umbilical cord. The privacy of the donor’s personal health information was protected. Human umbilical vein endothelial cells (HUVECs) were isolated according to previously described protocols^34^. HUVECs were cultured in Endothelial-Basal-Medium (EBM-2, Lonza) supplemented as recommended by the manufacturer. In all experiments, cells were grown until confluence and left for 2 additional days in Labtek 4-chambers (Fisher Scientific). Medium was replaced with fresh medium 16 h before infection, and with fresh non-supplemented medium 1 h before infection.

### P. aeruginosa strains and culture

*P. aeruginosa* CHA strain exhibits functional T3SS, pili and flagellum^35^. The CHAΔ*pilY1* strain was checked for defective twitching motility^33^. The *P. aeruginosa* strains used in this study were transformed with pIApX2-EGFP plasmid to allow fluorescence imaging. Clonal bacteria were grown in liquid LB medium at 37 °C under agitation until the cultures reached an optical density of 1.0, ensuring that they were in the exponential growth phase. Fifteen minutes prior to performing experiments, bacteria were diluted in non-supplemented EBM-2 at 37 °C.

### Videomicroscopy

Clean microscope coverslips (Marienfeld) or Labtek chambers (Nunc) containing cells were placed in an incubator equilibrated at 37 °C located on a DMIRE2 Leica Microscope controlled by HCImageLive software (Leica). Images from a 100X (N.A. 1.3–0.6) oil-immersion objective (HCX PL FLUOTAR, Leica) were recorded with a Hamamatsu ORCA-05G camera. A4 (Leica) and L5 filter cubes (Leica) were used for excitation and emission of Hoechst 33342 and EGFP fluorescence, respectively.

### Acquisition parameters

To observe *P. aeruginosa* motility on abiotic surfaces, a drop of culture medium (15 μL) containing bacteria was deposited on a microscope slide. To observe *P. aeruginosa* motility on cells, bacteria were added to the cell culture medium in Labtek chambers. The focus was set on the immobile bacteria attached to the surface. In both cases, non-motile bacteria were photobleached by a 5-min illumination before the start of filming. Swimming bacteria were then filmed at a rate of 28 images per second for 10 seconds. Then, Hoechst 33342 was added to the medium to allow imaging of nuclei in eukaryotic cells.

### Data analysis

The trajectories of bacteria were determined by following the centroid of the cell body as determined with half-pixel precision by the multi-particle tracking feature in SimplePCI and ImageJ softwares. Tracks from at least triplicate experiments were pooled. A 3-point central moving average filter was applied to smooth trajectories and reduce position noise. Only trajectories faster than 7 μm s^−1^ and showing a full straight-line distance longer than 4 μm, lasting 0.857 s (≥24 data points) were retained. This filtering allowed non-swimming and stuck bacteria to be rejected. A positive (negative) sign was assigned to CW (CCW) trajectory curvature, as viewed from the culture medium toward the cell surface. Each trajectory was characterized by K and V, the mean and median values of the temporal distributions of curvature (k) and speed (v), and termed CW or CCW according to the sign of K. Each trajectory was then segmented into sections if the instantaneous values of k and v for at least three consecutive points met the criteria defining one of the four types of section. The adjacent isolated points were assigned to the closest section. The section was called linear (L) when abs (θ), the change of angle (or turning speed) between two consecutive speed vectors, was ≤40 deg.s^−1^ and |K| was ≤0.025 μm^−1^; pauses (P) were defined when v/V was ≤0.66; curved clockwise (CW) sections, when 0 < K and |K| > 0.025 μm^−1^; and counter-clockwise (CCW) sections, when K < 0 and |K| > 0.025 μm^−1^. The frequencies of transitions were determined by calculating the number of sections of a given type per time unit.

### Confocal microscopy

Cells were imaged using a confocal spinning-disk inverted microscope (Nikon TI-E Eclipse) equipped with an Evolve EMCCD camera. Images were acquired using an illumination system from Roper Scientific (iLasPulsed) with a CFI Plan APO VC oil-immersion objective (60X, N.A 1.4). Z-series were generated using a motorized Z-piezo stage (ASI) by acquiring images with a step size of 0.4 μm.

### Statistics

Data were represented as box plots when the distribution was not Gaussian or when the variances were unequal. Solid circles represent the 5/95 percentiles. Statistical tests were performed using SigmaPlot software (Systat Software Inc.).

## Additional Information

**How to cite this article**: Golovkine, G. *et al*. Host cell surfaces induce a Type IV pili-dependent alteration of bacterial swimming. *Sci. Rep.* **6**, 38950; doi: 10.1038/srep38950 (2016).

**Publisher's note:** Springer Nature remains neutral with regard to jurisdictional claims in published maps and institutional affiliations.

## Supplementary Material

Supplementary Information

## Figures and Tables

**Table 1:** Characteristics of the three *P. aeruginosa* swimming classes.

	Class 1	Class 2	Class 3
Characteristics	Low speed Strong local curvature	Medium speed Weak curvature	High speed Very weak curvature
Interaction with surface	Strong	Medium	Weak or absent
