# Supplementary material for: Host cell surfaces induce a Type IV pili-dependent alteration of bacterial swimming
Source: Sci Rep. 2016 Dec 14;6:38950. doi: 10.1038/srep38950 (PMC5155295; doi:10.1038/srep38950)
Supplement: Supplementary Information [file srep38950-s1.pdf]

Supplementary Information

Host cell surfaces induce a type IV pili-dependent alteration of bacterial swimming

Guillaume Golovkine, Laurence Lemelle, Claire Burny, Cedric Vaillant, Jean-Francois Palierne, Christophe Place and Philippe Huber

Supplementary Table 1: Statistical performance and accuracy feature of « three-component gaussian mixture » models for the experimental speed distributions[a]

Strain/ Surface type	Model type	Percentages in G_1, G_2, G_3	Classification rate	BIC
WT/ Cell	P	30-40-30		
	M0	34-34-32	94	2541
	M1	40-33-27	87	2557
	M2	31-39-30	99	2588
	M3	40-33-27	86	2562
	M4	31-39-30	98	2573
WT/ Glass	P	13-43-44		
	M0	7-49-44	93	2639
	M1	29-33-38	79	2643
	M2	9-45-46	93	2746
	M3	28-34-38	80	2643
	M4	15-43-42	96	2662
Δpili / Cell	P	13-35-52		
	M0	13-40-47	95	2108
	M1	23-34-43	81	2112
	M2	14-38-48	95	2146
	M3	33-24-43	81	2112
	M4	14-35-51	98	2134
Δpili/ Glass	P	12-27-61		
	M0	11-23-66	93	2462
	M1	33-21-46	65	2643
	M2	11-25-64	95	2781
	M3	33-21-46	62	2466
	M4	13-27-60	99	2505

[a]The percentages studied in the manuscript and determined by a manual partition P of the experimental velocity (V) distributions in three classes delimitated by the two absolute V-values (20 $\mu m.s^{-1}$ and 50 $\mu m.s^{-1}$) are reported (highlighted in light grey). Every model is characterized by fixed relationships between the mean and the standard deviation (E_i, σ_i) of each ith gaussian component G_i ($i \in [1,3]$). M0 is a model where ($\sigma_1 \neq \sigma_2 \neq \sigma_3$). M1 is a model where ($\sigma_1 = \sigma_2 = \sigma_3$). M2 is a model where ($\sigma_1 = 3\ \mu m.s^{-1} << \sigma_2 = \sigma_3 = 9\ \mu m.s^{-1}$). M3 is a model where ($\sigma_1 = \sigma_2 = \sigma_3 = 9\ \mu m.s^{-1}$). M4 is a model where ($E_1 = 13\ \mu m.s^{-1}$, $\sigma_1 = 4\ \mu m.s^{-1}$), ($E_2 = 36\ \mu m.s^{-1}$, $\sigma_1 = 9\ \mu m.s^{-1}$) and ($E_3 = 66\ \mu m.s^{-1}$, $\sigma_3 = 9\ \mu m.s^{-1}$). Values in M4 are set from the manual partition P. For each model, each V-value was assigned to one of the three gaussians according to a MAP (Maximum A Posteriori)-based classification. The percentages of data assigned to every gaussian G_1-G_2-G_3 are reported. The classification rate (CR) was calculated as the sum of the number of concordant allocations compared to the partition P and according to a MAP-based classification divided by n. All the values were rounded to zero decimal places. The Bayesian information criterion values ($BIC = -2 \ln L + k \ln(n)$, where L is the maximized value of the likelihood function, n is the number of data points and k is the number of free parameters to be estimated) are reported to compare the lowest comparable CR values (involving possibly M0-M2-M4 models). The parsimonious model providing higher CR and lowest BIC values is systematically M4 (highlighted in dark grey), validating the partition P applied in the manuscript.

Supplementary Table 2: Radii of curvature of the curved sections[a]

Surface	Sense of curvature	Class 1	Class 2	Class 3
Glass	CW	0.28 ± 0.27 (152)	0.08 ± 0.05 (276)	0.05 ± 0.02 (115)
Glass	CCW	-0.29± 0.25 (132)	-0.07 ± 0.06 (212)	-0.06 ± 0.02 (201)
Cell	CW	0.28 ± 0.28 (394)	0.07 ± 0.06 (293)	0.04 ± 0.02 (74)
Cell	CCW	-0.28 ± 0.29 (404)	-0.07 ± 0.06 (253)	-0.06 ± 0.03 (110)

[a]The averaged radii of curvature ± SD of the curved sections of bacterial trajectories were calculated for each class and each surface type. The number of sections used are indicated between parenthesis.
